# Agreement between patients and mental healthcare providers on unmet care needs in child and adolescent psychiatry

**DOI:** 10.1007/s00127-020-01969-8

**Published:** 2020-09-30

**Authors:** Richard Vijverberg, Robert Ferdinand, Aartjan Beekman, Berno van Meijel

**Affiliations:** 1 Department of Child and Adolescent Psychiatry, GGZ Delfland Psychiatric Institute, GGZ Delfland, Delft, The Netherlands; 2 Inholland University of Applied Sciences, Amsterdam, The Netherlands; 3 Department of Psychiatry, Amsterdam Public Health Research Institute, Amsterdam UMC, VU Amsterdam, Amsterdam, The Netherlands; 4 Parnassia Psychiatric Institute, The Hague, The Netherlands

**Keywords:** Childhood, Agreement, Care needs, Intensity of care, Outpatient, Assertive Community Treatment

## Abstract

**Purpose:**

In mental health care, patients and their care providers may conceptualize the nature of the disorder and appropriate action in profoundly different ways. This may lead to dropout and lack of compliance with the treatments being provided, in particular in young patients with more severe disorders. This study provides detailed information about patient–provider (dis)agreement regarding the care needs of children and adolescents.

**Methods:**

We used the Camberwell Assessment of Need (CANSAS) to assess the met and unmet needs of 244 patients aged between 6 and 18 years. These needs were assessed from the perspectives of both patients and their care providers. Our primary outcome measure was agreement between the patient and care provider on unmet need. By comparing a general outpatient sample (*n* = 123) with a youth-ACT sample (*n* = 121), we were able to assess the influence of severity of psychiatric and psychosocial problems on the extent of agreement on patient’s unmet care needs.

**Results:**

In general, patients reported unmet care needs less often than care providers did. Patients and care providers had the lowest extents of agreement on unmet needs with regard to “mental health problems” (*k* = 0.113) and “information regarding diagnosis/treatment” (*k* = 0.171). Comparison of the two mental healthcare settings highlighted differences for three-quarters of the unmet care needs that were examined. Agreement was lower in the youth-ACT setting.

**Conclusions:**

Clarification of different views on patients’ unmet needs may help reduce nonattendance of appointments, noncompliance, or dropout. Routine assessment of patients’ and care providers’ perceptions of patients’ unmet care needs may also help provide information on areas of disagreement.

## Introduction

Over 40% of the children and adolescents who use mental healthcare terminate treatment prematurely, do not comply with treatment, or do not attend appointments regularly [1, 2]. Although this is a complex issue, one important factor may be that patients and mental healthcare providers do not agree on the nature of the problems and on the unmet care needs that need to be addressed during treatment [3–5]. Such a lack of agreement may lead to disagreement on the goals to be pursued and on appropriate treatment trajectories [6]. By negatively affecting attachment between patient and care provider, it may also affect their working relationship [7–9]. If the quality of the working alliance is poor, mental health problems may increase and functioning may deteriorate, ultimately leading to referral to a more intensive form of care [10–13].

We defined a “care need” as a physical, psychological, social, or environmental demand for aid, care or service intended to resolve a problem that a patient or his/her care provider perceived and expressed [14]. Care needs can be subdivided into (1) met needs, i.e., difficulties in a particular domain of functioning that are adequately taken care of; and (2) unmet needs, i.e., those for which a patient is not receiving the right care or the appropriate level of care [15].

Previous studies show that children and adolescents differ considerably from care providers with regard to the presence of psychiatric problems [16–18]. Care providers tend to report more problems than children/adolescents themselves. Higher levels of agreement were reported for externalizing problems (such as aggression and antisocial behavior) than for internalizing problems (such as sadness and anxiety) [16–18]. Although it is important during treatment to focus on psychiatric problems and related care needs, patients may also perceive care needs in other domains of functioning [19]. For this reason, a narrow focus on psychopathology-related care needs—and on possible disagreements between professionals and patients in this area—would not make it possible to fully understand patients’ unmet care needs. Overall, other studies in adults that had a broad focus on care needs in different areas of functioning found that psychiatric patients scored more unmet care needs than their care providers did [20–22], but that adult patients with severe psychiatric problems and psychosocial difficulties scored fewer needs [23].

There is currently little or no research on the extent to which children and adolescents agree or disagree with care providers on the broad range of met and unmet care needs. Therefore, the aim of our study was to obtain insight into the extent of agreement on these needs between the two groups. Further, we aimed to better understand whether the extent of agreement would differ between two setting with a different treatment intensity.

We had two a priori hypotheses: (1) that patients in the two settings would report less unmet care needs than their care providers; and (2) that we would find more disagreements between patients and care providers on the presence of patients’ unmet care needs in a youth-ACT setting—in other words, in a setting where patients had more severe psychiatric problems and psychosocial difficulties.

## Methods

### Design

In two different mental healthcare settings characterized by different severities of psychiatric problems and psychosocial difficulties, we used a cross-sectional design to compare the extent of agreement and disagreement between patients and mental healthcare providers on reported unmet care needs.

To increase our insight into the extent of agreement regarding these needs, we first established their frequencies in a specialized mental healthcare setting, approaching them from the perspectives of children and adolescents and also from those of care providers. We then examined the extent of agreement on these needs between the two groups. To better understand how the extent of agreement on patients' unmet care needs was influenced by the severity of psychiatric problems and by psychosocial difficulties, we compared unmet care needs between two treatment settings [24]. For this purpose, we included patients from a general outpatient mental healthcare setting and from youth-ACT, an Assertive Community Treatment setting. Youth-ACT is an intensive and outreaching mental healthcare service for children and adolescents with severe psychiatric and psychosocial problems [25–27].

### Setting

The study was performed in a specialized treatment center for child and adolescent psychiatry in the Netherlands. Patients and care providers were included from two settings that provided care for the same catchment area.

The first was a general outpatient treatment setting (with low to moderate treatment intensity), in which treatment was provided by a multidisciplinary team consisting of one child psychiatrist, six psychologists, and one nurse practitioner, who made diagnostic assessments and provided cognitive behavioral therapy, eye-movement desensitization, and reprocessing therapy (EMDR); family support; and pharmacological treatment.

The second was a youth-ACT setting (Assertive Community Treatment with high treatment intensity) consisting of one child psychiatrist, five psychologists, three nurse practitioners, and two mental health nurses. This team offered home-based outreach-oriented treatment to patients with more severe psychiatric and psychosocial problems. Care providers had small shared caseloads (< 15 patients) and provided outreaching case management, early intervention, cognitive behavioral therapy, EMDR, family support, and pharmacological treatment. The intensity of ACT treatment was scaled up or down according to the severity of a patient’s current psychiatric symptoms and psychosocial impairments.

### Participants

**Fig. 1:**
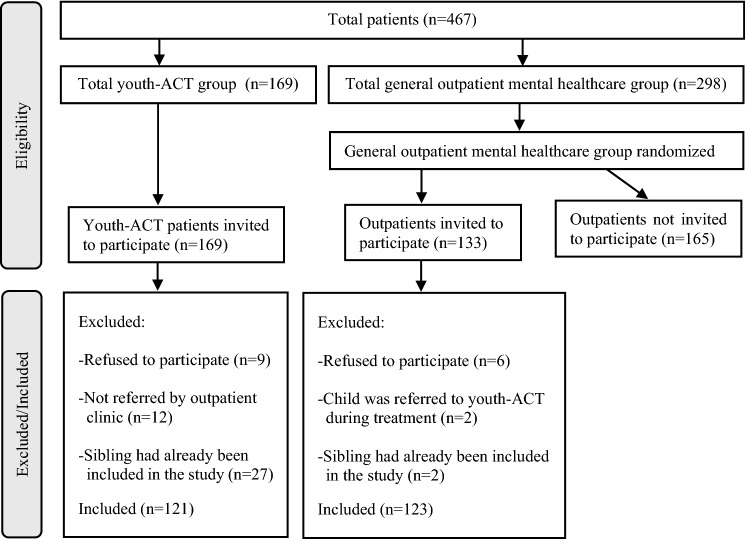
Participant flow diagram

Figure 1 presents the flowchart for inclusion. Participants, who were recruited between 2014 and 2016, were patients aged between 6 and 18 years. A total of 467 patients were considered for participation in the study. An initial random sample of 133 patients was selected from the general outpatient population of 298 patients. Next, 10 of these outpatients had to be excluded because they already had a sibling who participated in the study (*n* = 2), they refused to participate (*n* = 6), or were referred to the youth-ACT setting during the inclusion period (*n* = 2). For the youth-ACT sample, we initially selected all patients who were referred from a general outpatient setting to this ACT treatment setting during the inclusion period (*n* = 169). Thereafter, 48 ACT patients had to be excluded because they did not meet the inclusion criteria: 27 patients because their sibling was already included in the study, 12 patients were not referred to the ACT setting from the outpatient setting, but by the general practitioner instead, and 9 patients refused to participate. The final sample consisted of 244 patients: 123 in the outpatient sample and 121 in the youth-ACT sample.

### Ethical approval

The study was reviewed and approved by the Medical Ethical Committee at VU University Medical Centre Amsterdam (protocol no. 2015.245) and by the Scientific Committee at the EMGO^+^ Institute for Health and Care Research Amsterdam. Approval was also provided by the local scientific review board of the participating mental health institution.

Separately, children/adolescent participants, and their parents received written and oral information on the research project. In accordance with prevailing Dutch legislation, written consent from parents and/or children/adolescents was obtained as follows: (i) if children younger were aged less than 12, only parents were asked for consent for; (ii) if children were aged between 12 and 16, parents and children were both asked for consent; and (iii) if adolescents were 16 years or older, informed consent was obtained from them alone.

### Measurement instruments

The Demographic Information Questionnaire (DEMOG) was used to measure the following four demographic characteristics of each child or adolescent: (1) age, (2) gender, (3) country of birth, and (4) living in a single-parent family or a two-parent family [28].

The Neuropsychiatric Interview for Children and Adolescent (MINI-KID) was used to assess patients’ psychiatric diagnoses [29], which were supplemented with clinical diagnoses that were not included in the MINI-KID [30].

The Camberwell Assessment of Need Short Appraisal Schedule (CANSAS) was used to assess the patient’s care needs as they were perceived both by the child/adolescent and by the mental healthcare provider [15]. CANSAS covers 23 items, each with three response options: (1) no need (= no problem); (2) met need (= difficulties in a particular domain of functioning that is receiving suitable assessment or intervention); (3) and unmet need (= difficulties in a particular domain of functioning that requires further assessment or is not receiving the right care or an appropriate level of care) [31]. To categorize CANSAS items (see Tables 2, 3), we used the following three International Classification of Functioning, Disability and Health (ICF) health and health-related domains: (i) physical and mental functions, (ii) performance of daily activities, and (iii) participation in the community [19, 32, 33].

### Data analysis

The participants’ demographics were analyzed using descriptive statistics, first for the entire sample and then separately for the two subgroups (general outpatient setting and the youth-ACT setting). Subgroup differences regarding age, gender, country of birth, general functioning, and living situation were analyzed using the *t* test for continuous variables, or using the Chi-square test with Yates continuity correction (χ^2^ test) for categorical variables. As an alternative to the Chi-square test, Fisher’s exact test was computed if the number in at least one of the cells of the categorical variable was lower than 5 [34].

To analyze the extent of agreement between patients and care providers at item level, Cohen’s kappa coefficients were computed for the overall sample and then separately for the two treatment settings. On the basis of the Cohen’s kappa values, the extent of agreement was categorized as follows: poor (≤ 0.20); fair (0.21–0.40); moderate (0.41–0.60); good (0.61–0.80); or very good (≥ 0.81–1.00) [35].

To determine whether patient and care provider agreed or disagreed on the presence of an unmet need, the following calculation was made for both settings at CANSAS-item level: only the patient reported an unmet care need (P > CP); patient and care provider both reported the presence or absence of an unmet care need (P = CP); or only the care provider reported an unmet care need (P < CP) (see Table 3). Frequencies of agreement or disagreement on unmet care needs between patients and care providers were calculated for the two treatment settings, and subgroup differences between these settings were analyzed using Fisher’s exact test [34]. For all calculations, a value below *p* < 0.05 was considered to be statistically significant [35].

## Results

### Characteristics of the study sample

Table 1 shows the demographic characteristics of our two study samples. As would be expected, patients in the regular outpatient sample had a significantly higher score for overall functioning (GAF) than those in the youth-ACT sample (mean = 55.0, sd = 5.4 vs. mean = 45.9, sd = 8.0; *p* < 0.05).

**Table 1:** Sample characteristics of the child or adolescent who received treatment

	Outpatient	Youth-ACT	*t* test/corrected χ^2^ test (two-sided) (df = 1)/Fisher’s exact test	*p*
	Child (*n* = 123)	Child (*n* = 121)		
Age (sd)				
Total mean	12.4 (3.2)	13.0 (3.1)	− 1.584^t^	0.439
Range	6–17	6–18		
Girls mean	12.6 (3.4)	13.8 (2.8)	− 0.733^t^	0.054
Range	6–17	6–18		
Boys mean	11.1 (2.9)	12.5 (3.2)	− 1.876^t^	0.339
Range	6–17	6–17		
Gender				
Girls	45.5%	42.9%	1.001^χ2^	0.317
Boys	54.5%	57.1%		
Country of birth				
The Netherlands	96.7%	95.0%		0.536^FE^
Other	3.3%	5.0%		
Clinical diagnoses				
Mood	6.5%	37.2%	31.999^χ2^	0.000
Anxiety	31.7%	42.1%	2.426^χ2^	0.119
Behavior	12.2%	29,8%	10.335^χ2^	0.001
Psychotic	0.0%	4.1%		0.029^FE^
ASD	11.4%	39.7%	24.281^χ2^	0.000
ADHD	43.1%	43.4%	0.000^χ2^	1.000
Somatoform	0.8%	13.2%		0.000^FE^
Drugs/alcohol	0.0%	3.3%		0.059^FE^
Personality	0.0%	2.9%		0.007^FE^
Intelligence below or well below average	3.3%	8.3%		0.106^FE^
Other	0.8%	3.3%		0.211^FE^
GAF-score (sd)				
Mean	55.0 (5.4)	45.9 (8.0)	10.460^t^	0.000
Range	45–75	15–60		
Living situation				
Single parent	26.2%	42.3%	6.624^χ2^	0.010
Two parents	73.8%	57.7%		

An independent sample *t* test was performed to compare the mean score between the outpatient and youth-ACT samples with respect to the continuous variable. The χ^2^ test with a continuity correction was used to test the difference between the outpatient and youth-ACT sample with regard to a categorical variable with df = 1

*ADHD* attention deficit hyperactivity disorder, *ASD* Autism spectrum disorder, *GAF* general assessment of functioning, *df* degree of freedom, *n* number of included patients, *p* *p* value; a value below 0.05 is considered to be statistically significant, *FE* Fisher’s exact test was performed because the number in at least one of the cells in the child or care provider sample was < 5, *sd* standard deviation

In the subsample of youth-ACT patients significantly higher frequencies were found for ASD (39.7%), mood (37.2%), behavior (29.8%), somatoform (13.2%), and personality (2.9%), indicating higher levels of comorbidity in patients treated in youth-ACT. Significantly more of those receiving ACT treatment reported growing up in a single-parent family (42.3%) than those receiving regular outpatient treatment (26.2%; *p* < 0.05). There were no significant differences between the two samples regarding age, gender, and country of birth.

### Agreement between patients and care providers in the overall sample

Table 2 shows the kappa coefficients (*k*) that were calculated to determine the agreement between patients and care providers in the overall sample at CANSAS item level. In general, patients reported unmet care needs less than care providers did. Below, our results are presented using the ICF domains, but, for purpose of conciseness, we now report only the results whose frequency in at least one of the two settings (outpatient or youth-ACT) was higher than 10%.

**Table 2:** Unmet needs overall sample

Unmet needs domains	Child		Care provider		Level of agreement overall sample		Corrected χ^2^-test (two-sided) (df = 1)/Fisher’s exact test	*p*
	(*n* = 244)		(*n* = 245)					
	*N*	%	*N*	%	*k*	95% CI		
Physical and mental functions								
Mental health problems (not psychotic)	156	63.9	229	93.5	0.113	0.023 to 0.203	61.93	0.000
Danger to others	19	7.8	49	20.0	0.277	0.126 to 0.428	14.23	0.000
Danger to themselves	18	7.4	61	24.9	0.271	0.140 to 0.400	26.43	0.000
Psychotic symptoms	19	7.8	26	10.6	0.438	0.244 to 0.632	0.85	0.355
Information regarding diagnosis/treatment	133	54.5	202	82.4	0.171	0.069 to 0.273	42.95	0.000
Physical handicap or disease	27	11.1	35	14.3	0.570	0.413 to 0.727	0.87	0.350
Medication side effects	26	10.7	23	9.4	0.660	0.499 to 0.821	0.10	0.752
Drugs misuse/alcohol abuse	5	2.0	11	4.5	0.326	− 0.162 to 0.814	1.59	0.207
Food (qualitative or quantitative)	19	7.8	31	12.7	0.380	0.198 to 0.562	2.65	0.104
Performance of daily activities								
Reading/writing skills at expected grade level	34	13.4	38	15.5	0.687	0.556 to 0.818	0.13	0.716
Handling money	21	8.6	30	12.2	0.411	0.231 to 0.591	1.37	0.243
Self-care abilities (age-related)	22	9.0	53	21.6	0.328	0.183 to 0.473	14.03	0.000
Paid job (included side-jobs)	22	9.0	36	14.7	0.534	0.371 to 0.697	3.25	0.072
Cleaning up room (or bedroom)	15	6.1	32	13.1	0.233	0.056 to 0.409	5.95	0.015
Caring for someone else (family member or pet)	2	0.8	7	2.9	− 0.130	− 0.144 to − 0.116	FE	0.176
Participation in the community								
Regular/suitable school or other daytime activities	56	23.0	105	42.9	0.437	0.331 to 0.543	21.04	0.000
Making and/or keeping friends	64	26.2	137	55.9	0.299	0.201 to 0.397	43.29	0.000
Future prospects (opportunities/chances of a successful and prosperous life)	82	33.6	160	65.3	0.364	0.274 to 0.454	47.88	0.000
Access to (public) transport	13	5.3	23	9.4	0.479	0.269 to 0.689	2.39	0.122
Housing	11	4.5	21	8.6	0.535	0.323 to 0.747	2.67	0.102
Access to modern tools of communication	7	2.9	5	2.0	0.488	0.137 to 0.839	0.09	0.765
Intimate relations	18	7.4	36	14.7	0.561	0.398 to 0.724	5.94	0.015
Sexuality	5	2.0	34	13.9	0.181	0.020 to 0.342	21.72	0.000

The χ^2^ test with a continuity correction was performed because df = 1. Fisher’s exact test was performed if the number in at least one of the cells in the child or care-provider sample was < 5

*n* number of included patients, *N* number of reported unmet needs, *k* Cohen’s kappa poor (≤ 0.20); fair (0.21–0.40); moderate (0.41–0.60); good (0.61–0.80); or very good (≥ 0.81–1.00),* FE* Fisher’s exact test, *p* *p* value; a value below 0.05 is considered to be statistically significant

#### Physical and mental functions

Agreement between patients and care providers on unmet needs for “mental health problems”—the most reported unmet care need—was poor (*k* = 0.113), with the scores between patients (63.9%) and care providers (93.5%) differing significantly (*p* < 0.05). Agreement on unmet care need for “information regarding diagnosis/treatment” was also poor (*k* = 0.171), with patients (54.5%) reporting unmet needs significantly less than care providers (82.4%; *p* < 0.05). Agreement on “danger to others” was fair (*k* = 0.277), with patients reporting significantly fewer unmet needs (7.8%) than care providers (20.0%; *p* < 0.05). Agreement was also fair with regard to “danger to themselves” (*k* = 0.271), with patients reporting 7.4% and providers reporting 24.9% (*p* < 0.05). Agreement on unmet care needs related to “psychotic symptoms” was moderate (*k* = 0.438), with no significant differences between the two groups (patients 7.8% vs. providers 10.6%). Agreement for “medication side effects” was good (*k* = 0.660; patients 10.7% vs. providers 9.4%; ns). Agreement on physical functions was moderate for unmet needs related to “physical handicap or disease” (*k* = 0.570; patients 11.1% vs. providers 14.3%; ns). Finally, agreement on “quality or quantity of food” was only fair (*k* = 0.380), with no significant differences between the two groups (patients 7.8% vs. providers 12.7%).

#### Performance of daily activities

Patient–provider agreement on abilities of self-care (e.g., daily hygiene and oral health) as an unmet care was fair (*k* = 0.328); the difference between patients (9%) and care providers (21.6%) was statistically significant (*p* < 0.05). Agreement on “cleaning up room or bedroom” was also fair (*k* = 0.233; patients 6.1% vs. providers 13.1%; *p* < 0.05). Agreement on “handling money” was moderate (*k* = 0.411; patients 8.6% vs. providers 12.2%), as was agreement on “paid job or side job” (*k* = 0.534; patients 9.0% vs. providers 14.7%), with no significant difference between the two groups. Agreement on unmet needs regarding “reading and writing skills at expected grade level” was good (*k* = 0.687), with no significant difference between patients (13.4%) and care providers (15.5%).

#### Participation in the community

Agreement on “future prospects” (e.g., opportunities/changes for a successful and prosperous life)—a frequently reported unmet need—was fair (*k* = 0.346; patients 33.6% vs. providers 65.3%, *p* < 0.05). With regard to “making and/or keeping friends” as an unmet care need, patients, and care providers reported significantly differently, leading to only fair agreement (*k* = 0.299; 26.2% vs. 55.9%, respectively; *p* < 0.05). Agreement on unmet needs related to “regular/suitable school or other daytime activities” (e.g., practicing a hobby/sport) were moderate (*k* = 0.437), with significant differences between patients (23.0%) and providers (42.9%; *p* < 0.05). Patient–provider agreement for unmet care needs related to “intimate relations” was moderate (*k* = 0.561), with patients (7.4%) reporting significantly fewer unmet care needs related to “intimate relations” than care providers (14.7%; *p* < 0.05). Agreement on the presence of unmet needs related to “sexuality” was only poor (*k* = 0.181; patients 2.0% vs. providers 13.9%; *p* < 0.05).

### Comparison of agreement between outpatient clinics and youth-ACT

Comparison of youth-ACT and outpatient clinics showed significant differences between the two settings for three-quarters of the unmet care needs that were investigated (see Table 3). Compared with their peers in the outpatient setting, youth-ACT patients agreed less with their care providers (P = CP) on the presence or absence of an unmet need for care. If there was disagreement, patients, unlike their care provider, did not usually report an unmet care need (P < CP). Below, for reasons of brevity, we highlight solely results of P < CP whose frequency in at least one of the two settings was higher than 10%.

**Table 3:** Unmet needs disagreement and agreement child–care provider

	Outpatient (*n* = 123)						Youth-ACT (*n* = 120)						
	P > CP	%	P = CP	%	P < CP	%	P > CP	%	P = CP	%	P < CP	%	*p* (Fisher’s exact test)
Physical and mental functions													
Mental health problems (not psychotic)	2	1.6	86	69.9	35	28.5	3	2.5	75	62.5	42	35.0	0.522
Danger to others	1	0.8	113	91.9	9	7.3	3	2.5	79	65.8	38	31.7	0.000
Danger to themselves	3	2.4	116	94.3	4	3.3	4	3.3	84	70.0	32	26.7	0.000
Psychotic symptoms	2	1.6	115	93.5	6	4.9	6	5.0	105	87.5	9	7.5	0.219
Information regarding diagnosis/treatment	3	2.4	88	71.5	32	26.0	10	8.3	60	50.0	50	41.7	0.001
Physical handicap or disease	1	0.8	120	97.6	2	1.6	7	5.8	100	83.3	13	10.8	0.000
Medication side effects	1	0.8	121	98.4	1	0.8	8	6.7	107	89.2	5	4.2	0.008
Drugs misuse/alcohol abuse	–	–	121	98.4	2	1.6	3	2.5	111	92.5	6	5.0	0.054
Food (qualitative or quantitative)	1	0.8	119	96.7	3	2.4	7	5.8	96	80.0	17	14.2	0.000
Performance of daily activities													
Reading/writing skills at expected grade level	3	2.4	117	95.1	3	2.4	5	4.2	107	89.2	8	6.7	0.185
Handling money	5	4.1	115	93.5	3	2.4	4	3.3	101	84.2	15	12.5	0.009
Self-care abilities (age-related)	–	–	115	93.5	8	6.5	5	4.2	85	70.8	30	25.0	0.000
Paid job (included side-jobs)	2	1.6	119	96.7	2	1.6	3	2.5	100	84.2	17	14.2	0.000
Cleaning up room (or bedroom)	3	2.4	118	95.9	2	1.6	5	4.2	92	76.7	23	19.2	0.000
Caring for someone else (family member or pet)	–	–	122	99.2	1	0.8	2	1.7	112	93.3	6	5.0	0.026
Participation in the community													
Regular/suitable school or other daytime activities	–	–	113	91.9	10	8.1	7	5.8	67	55.8	46	38.3	0.000
Making and/or keeping friends	2	1.6	101	82.1	20	16.3	7	5.8	52	43.3	61	50.8	0.000
Future prospects (opportunities/chances of a successful and prosperous life)	2	1.6	94	76.4	27	22.0	2	1.6	64	53.3	54	45.0	0.000
Access to (public) transport	1	0.8	121	98.4	1	0.8	4	3.3	105	87.5	11	9.2	0.001
Housing	–	–	121	98.4	2	1.6	2	1.7	108	90.0	10	8.3	0.008
Access to modern tools of communication	–	–	123	100	–	–	4	3.3	114	95.0	2	1.7	0.095
Intimate relations	–	–	122	99.2	1	0.8	2	1.7	100	83.3	18	15.0	0.000
Sexuality	–	–	119	96.7	4	3.3	1	0.8	94	78.3	25	20.8	0.000

Fisher’s exact test was performed because the number in at least one of the cells in the child or care provider sample was < 5

*n* number of included patients, *P > CP* only the patient reported a unmet care need, *P = CP* patient and care provider both reported the presence or absence of an unmet care need, *P < CP* only the care provider reported a unmet care need, *p* *p* value; a value below 0.05 is considered to be statistically significant

#### Physical and mental functions

As Table 3 shows, relative to those in the outpatient setting, patients in the youth-ACT setting reported that they had no unmet needs with regard to “information regarding treatment and/or diagnosis” (P < CP 41.7%) significantly more than the care provider did (P < CP 26.0%; *p* < 0.05).

With regard to unmet care needs for “danger to others” and “danger to themselves,” patients and care providers (P < CP 31.7% and P < CP 26.7%, respectively) in the youth-ACT sample disagreed significantly more than patients and their care providers (P < CP 7.3% and P < CP 3.3%, respectively; *p* < 0.05) in the outpatient sample.

With regard to “quality and/or quantity of food”, there were significant differences (*p* < 0.05) between the two settings, with ACT patients (P < CP 14.2%) disagreements more on this item than outpatients (P < CP 2.4%).

With respect to the unmet need for “mental health problems”, there were no significant differences between patient–provider disagreements in the ACT setting (P < CP 35.0%) and those in the regular outpatient setting (P < CP 28.5%; ns).

#### Performance of daily activities

With regard to “abilities for self-care”, patients receiving youth-ACT treatment reported unmet care needs significantly less than their care providers (P < CP 25.0%), and significantly less than outpatients (P < CP 6.5%; *p* < 0.05). There were also significant differences (*p* < 0.05) between the youth-ACT sample and outpatient sample with regard to three unmet care needs: “cleaning-up room or bedroom” (youth-ACT P < CP 19.2% vs. outpatients P < CP 1.6%); “paid job or side job” (youth-ACT P < CP 14.2% vs. outpatients, P < CP 1.6%); and “handling money” (youth-ACT P < CP 12.5% vs. outpatients P < CP 2.4%).

#### Participation in the community

With regard to friendship-related unmet needs, patients in the youth-ACT sample scored significantly less than their care providers did (P < CP 50.8%), and significantly less than those receiving outpatient care (P < CP 16.3%; *p* < 0.05). Youth-ACT patients had significantly more patient–provider disagreements (*p* < 0.05) than outpatients with regard to unmet needs pertaining “future prospects” (youth ACT P < CP 45.0% vs. outpatients P < CP 22.0%); “regular/suitable school or other daytime activities” (youth-ACT vs. outpatients P < CP 38.3%, P < CP 8.1%); “sexuality” (youth-ACT P < CP 20.8% vs. outpatients P < CP 3.3%); and “intimate relations” (youth-ACT P < CP 15.0% vs. outpatients P < CP 0.8%).

## Discussion

This study is based on the assumption that agreement among patients and care providers on relevant care needs is a prerequisite not only for efficient and effective collaboration, but also for treatment adherence and treatment outcomes. Although such agreement may be even more relevant among young patients than among adults, it has not, to our knowledge, been studied systematically.

In general, care needs (met and unmet) can be studied on different levels, e.g. (i) the problems experienced by the client; (ii) the interventions required to alleviate or limit these problems; (iii) the services required to provide these interventions. A specific problem can be solved (and related care needs can be met) by several different interventions, which can be applied by different types of services. Since the presence of a problem may require one or more interventions to ameliorate these problems, some authors suggested that needs should not only be assessed at the problem level, but also at the intervention level [36]. In this study we have focused in the first instance on the problem level because that is where the treatment process starts, namely with the initial question: do the patient and/or practitioner think the patient has a problem for which care is needed or not?

### Agreement between patients and care providers in the overall sample

In general, agreement between patients and care providers with regard to patients' unmet care needs was low (see Table 2). While 23 unmet care needs were investigated, we found poor agreement for four, fair agreement for eight, moderate agreement for nine, and good agreement for only two. The lowest level of agreement was found for “mental health problems” and “information regarding diagnosis and/or treatment.” This is remarkable, as these two care needs are key topics during psychiatric treatment.

Overall, in line with our first hypothesis, patients reported fewer unmet care needs than their care providers (P < CP). The first possible explanation for this is that the care provider obtained information not only from the child, but also from the parents, whose views on appropriate care needs often differ from those of their children [37–39]. A second possible explanation lies in the rather self-evident fact that while patients tend to make personal statements, care providers’ statements also reflect a professional judgement [40].

### Agreement in youth-ACT versus outpatient setting

Comparison of the youth-ACT setting with the general outpatient treatment setting showed significant differences with regard to three-quarters of the unmet care needs (see Table 3). In both settings, fewer patients than care providers reported unmet care needs (P < CP). The extent of disagreement was higher in the youth-ACT setting, which was in line with our second hypothesis. A possible explanation for this is that patients in the youth-ACT sample had more severe psychiatric problems [27]. Such patients are more likely to report fewer problems and needs—because they may be less aware of existing problems, are sometimes less willing to seek solutions, or believe that persisting problems cannot be resolved [23, 41, 42]. Higher frequencies of ASD (39.7%), mood (37.2%), behavior (29.8%) and somatoform (13.2%) were found in the youth-ACT sample (see Table 1). Overall, in the ACT sample more comorbidity was assessed, which supports the hypothesis that the patients in this sample had more severe psychopathology.

Another explanation why youth-ACT patients disagreed more than outpatients may be that more of these patients came from multiproblem families [27]. When a patient lives in an environment that is potentially harmful to his or her development, care providers tend to report more unmet care needs [43]. On the other hand, patients may be tempted to report unmet care needs less often when they have grown up in living situations in which they have become accustomed to the presence of problems. In contrast, care providers, who have more distance, do identify problems [44, 45].

A third explanation is that, due to the home visits ACT care providers made during the intake phase, when they observed patients in their own living environment, ACT care providers depended less than outpatient care providers on information provided by the patient to form a picture of his or her unmet care needs.

### Implications for clinical practice and research

For clinical practice, the key to preventing noncompliance, nonattendance at appointments, and dropout may be in care providers’ awareness that their view of a patient’s unmet care needs often differs from that of the patient. We therefore recommend care providers—particularly those in youth-ACT settings or other intensive treatment settings—to routinely assess a child’s perceived care needs and compare them with their own perceptions of unmet care needs. Given the higher levels of comorbidity in the ACT sample, the examination of specific care needs related to this comorbidity should receive special attention in clinical practice. By sharing information on their perceptions of such needs, and by being explicit about the areas in which they disagree, patients and care providers can engage in a process of decision making that makes it possible to formulate goals and interventions on which they can then collaborate. Unmet care needs on which there is no agreement can be assessed according to their urgency; it may prove possible to postpone further attention to them until a later treatment phase.

We studied unmet care needs at the problem level, and investigated agreement regarding need for care, irrespectively of the type of intervention or services needed. In the future, it may be interesting to investigate whether different informants have a common view on the interventions required, once they agree on the problems that need to be addressed during treatment. Future research could also address the impact of improving agreement between patients and care providers with regard to unmet care needs on compliance with treatment and its outcomes.

### Strengths and limitations

This is the first study to provide detailed information about patient–provider (dis)agreement regarding the care needs of children and adolescents who have been referred to general outpatient care and youth-ACT. This is a strength because children and adolescents with different severity levels of psychiatric problems were studied, which supported the generalizability of findings.

A limitation of the study is its cross-sectional design, which prevented us from identifying causes of disagreements between patients and care providers on patients’ unmet care needs [46].

## Conclusions

We found that patients and care providers often disagreed on patients’ care needs, particularly in a youth-ACT treatment setting. Clarifying different views on patient’s unmet care needs may help to reduce nonattendance of appointments and early termination of treatment. Similarly, if patients and care providers systematically assessed patients’ unmet care needs, useful information may be provided on areas of disagreement. Future research should show whether better treatment outcomes would be produced by an approach focused on obtaining a shared view on unmet care needs.

## Data Availability

As the participants have not granted permission for data sharing, the data are not available.

## Abbreviations

ACT: Assertive Community Treatment
CANSAS: Camberwell Assessment of Need Short Appraisal Schedule
df: Degrees of freedom
CP: Care provider
EMDR: Eye-movement desensitization and reprocessing therapy
FE: Fisher’s exact test
GAF score: Global Assessment of Functioning score
ICF: International Classification of Functioning and Disability
K: Cohen’s kappa coefficient
MINI-KID: MINI International Neuropsychiatric Interview for Children and Adolescents
ns: Not significant
P: Patient
SD: Standard deviation
SPSS: Statistical Package for the Social Sciences
χ^2^ test: Chi-square test
